# Thyroid axis hormones and anthropometric recovery of children/adolescents with overweight/obesity: A scoping review

**DOI:** 10.3389/fnut.2022.1040167

**Published:** 2023-01-13

**Authors:** Carlos Ramos Urrea, Amanda Paula Pedroso, Fernanda Thomazini, Andreia Cristina Feitosa do Carmo, Mônica Marques Telles, Ana Lydia Sawaya, Maria do Carmo Pinho Franco, Eliane Beraldi Ribeiro

**Affiliations:** ^1^Department of Physiology, Escola Paulista de Medicina, Universidade Federal de São Paulo, São Paulo, Brazil; ^2^Escola Paulista de Medicina, Campus São Paulo Library, Universidade Federal de São Paulo, São Paulo, Brazil

**Keywords:** thyroid hormones, multidisciplinary intervention, obesity, children, adolescent, recovery

## Abstract

**Introduction:**

Thyroid hormones exert multiple physiological effects essential to the maintenance of basal metabolic rate (BMR), adaptive thermogenesis, fat metabolism, growth, and appetite. The links between obesity and the hormones of the thyroid axis, i.e., triiodothyronine (T3), thyroxine (T4), and thyrotropin (TSH), are still controversial, especially when considering children and adolescents. This population has high rates of overweight and obesity and several treatment approaches, including nutritional, psychological, and physical exercise interventions have been used. Understanding the importance of the hormones of the thyroid axis in the recovery from overweight and obesity may help directing measures to the maintenance of a healthy body composition. The present scoping review was carried out to analyze studies evaluating these hormonal levels throughout interventions directed at treating overweight and obesity in children and adolescents. The main purpose was to ascertain whether the hormones levels vary during weight loss.

**Methods:**

We selected for analysis 19 studies published between 1999 and 2022.

**Results:**

Most of the studies showed that changes in different anthropometric indicators, in response to the multidisciplinary interventions, correlated positively with free T3 (fT3), total T3 (TT3), and TSH. With respect to free T4 (fT4) and total T4 (TT4).

**Discussion:**

The most common finding was of unchanged levels and, hence, no significant association with weight loss. Moreover, thyroxine supplementation has failed to affect the response to the interventions. Further studies are necessary to elucidate the relevance of the variations in hormone levels to the establishment of overweight/obesity and to the recovery from these conditions in children/adolescents.

**Systematic review registration:**

https://www.crd.york.ac.uk/prospero/, identifier CRD42020203359.

## 1. Introduction

Globally, obesity is a well-recognized public health problem affecting both adults and children (1). In children/adolescents, the prevalence of overweight/obesity is high (2) and associates with increased risk to develop diabetes and other co-morbidities (3).

The pathophysiology of overweight/obesity includes genetic, environmental, behavioral, metabolic, psychological factors, and hormonal factors. The thyroid hormones exert multiple physiological effects essential to the maintenance of basal metabolic rate (BMR), adaptive thermogenesis, fat metabolism, growth, and appetite (4). The participation of the levels of the hormones of the thyroid axis, i.e., thyrotropin-releasing hormone (TRH), thyrotropin (TSH), triiodothyronine (T3), and thyroxine (T4), and has been studied with no conclusive results, especially when considering children and adolescents. They have indicated either that thyroid-hormones resistance is a causal factor of obesity or that elevated hormone levels may represent an adaptive response to obesity (5).

The treatment of children and adolescents with overweight or obesity is a very relevant issue, and several approaches, including nutritional, psychological, and physical exercise interventions have been used (6). Understanding the importance of the hormones of the thyroid axis in the recovery from overweight and obesity may help directing measures to the maintenance of a healthy body composition.

The present scoping review was carried out to analyze studies evaluating these hormonal levels throughout interventions directed at treating overweight and obesity in children and adolescents.

## 2. Materials and methods

This scoping review was registered on the International Prospective Register of Systematic Reviews (PROSPERO, CRD42020203359) and performed in accordance with the recommendations of Preferred Reporting Items for Systematic Reviews and Meta-Analyses extension for Scoping Reviews (PRISMA-ScR).

### 2.1. Eligibility criteria

We included original articles published in peer-reviewed journals, written in English, Portuguese, or Spanish, that had children and/or adolescents with overweight or obesity as participants and that performed some intervention for weight management, including, nutritional and/or psychological, and/or medical, and/or exercise.

The exclusion criteria were studies in animals or adults, use of growth hormone or steroid hormones, diagnostic of thyroid, kidney, heart, or neurological illness. The types of studies included were clinical trials and longitudinal studies. Review articles were excluded.

### 2.2. Literature search

Data collection and analysis were performed in January 2022. Electronic searches were conducted using the following databases: MEDLINE via Pubmed, Latin American and Caribbean Literature in Health Sciences (LILACS), Scopus (Elsevier) and Cochrane Library. All articles appearing in the searches were included, with no pre-determined period.

The following descriptors were extracted from the Health Science Descriptors database: obesity, overweight, obese, excess weight, weight gain, malnutrition, thyroid hormones, thyroid concentrations, thyroid-stimulating hormone, triiodothyronine, thyroxine, thyroid gland, child, children, adolescents, humans. The development of the search strategy followed the recommendations of the checklist Peer Review of Electronic Search Strategies (PRESS) (7).

### 2.3. Study selection and appraisal

Three authors performed independent selection and analysis of the studies, using the Rayyan tool. The first selection was based on the title and summary of the studies. Duplicates and articles whose full texts were not available were excluded. Conflicts were resolved by consensus. After selection according to the inclusion criteria, the 3 authors independently analyzed the full texts to identify the relevant outcomes.

### 2.4. Data extraction and synthesis of results

To characterize the findings, the following variables were considered: age, sex, type of intervention, effect of the intervention on body composition and on hormone levels, both at baseline and after the intervention. The articles were grouped by type of comparisons performed (intra-group or between obese and eutrophic). Two studies involving thyroxine supplementation constituted a third category.

## 3. Results

**FIGURE 1:**
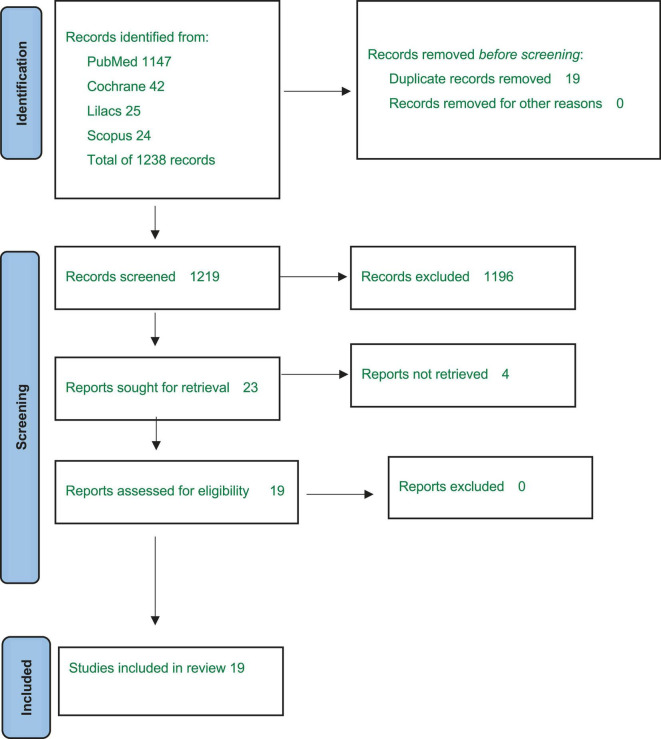
Flow diagram of the studies’ selection.

A total of 1,219 articles were screened, leading to 23 eligible articles, of which 4 were excluded due to absence of full texts. Nineteen articles were thus included in the qualitative analysis. Figure 1 describes the selection process and Supplementary Table 1 shows the results of each selected articles.

### 3.1. Description of the included studies

The literature search identified 1219 references (Figure 1) and 19 articles met all the inclusion criteria.

Supplementary Table 1 summarizes the characteristics of the studies included in the analysis. There are studies performed with subjects from Germany (15, 17–19), Italy (8, 11,20), France (9, 13), Israel (21, 22), Turkey (14, 23), Netherlands (24), Switzerland (10), United States (16), Brazil (12), Tunisia (25), and India (26).

Thirteen studies analyzed only obese children and adolescents (8, 10, 11, 13–19, 21, 22, 26) while the other 6 studies targeted both overweight and obese children and/or adolescents (9, 12, 20, 23–25).

In 4 studies (13–16) the baseline hormonal values were not informed but the authors reported the changes induced by the interventions, hence they were included in the analysis.

Table 1 describes the correlations between the thyroid hormonal axis and body composition parameters in the 9 studies that performed this calculation. At baseline, body weight was negatively correlated with fT3 (10), the percentage of fat mass was positively correlated with fT4 (10), and BMI was positively correlated with TSH (8, 9, 11) and fT3 (8, 12). After intervention, BMI correlated positively with fT3, and/or TSH, TT3, and TT4 (11, 12, 14–16). When considering the baseline/after intervention changes, the authors reported positive correlations of BMI and TT3 (13, 15, 16), fT3 (10, 12, 14), TT4 (15), and TSH (11, 14, 16). Also, fT3 correlated positively with body weight, fat mass, and percentage body fat (10). Negative correlations were seen between lean body mass and fT3 and percentage body fat and fT4 (10).

**TABLE 1:** Correlations between anthropometric recovery and hormonal levels.

References/Correlation test	Significant correlations	
	**Baseline**	**After intervention**
Marras et al. (20) Pearson correlation	TSH and fT3 correlated positively with BMI-SDS	
Bouglé et al. (18) Simple correlations	TSH correlated positively with BMI *Z*-score	
Aeberli et al. (13) Pearson analysis	Body weight correlated negatively with fT3 Percentage body fat correlated positively with fT4	Delta fT3 correlated positively with body weight, BMI-SDS, fat mass, and percentage body fat and negatively with lean body mass. Delta fT4 correlated negatively with percentage body fat
Licenziati et al. (17) Bivariate correlation	TSH correlated positively with BMI-SDS	TSH correlated positively with BMI-SDS
Martins et al. (8) Pearson and Spearman correlations	fT3 correlated positively with BMI/Age Z-score	fT3 correlated positively with BMI/Age *Z*-score
Kiortsis et al. (11) Pearson correlation	TT3 correlated positively with BMI	
Bas et al. (15) Pearson correlation		TSH and fT3 correlated positively with BMI *Z*-score
Reinehr and Andler (23) Pearson correlation		TT3 and TT4 correlated positively with BMI *Z*-score
Butte et al. (24) Pearson correlation		TT3 and TSH correlated positively with BMI

### 3.2. Interventions

The duration of the interventions varied from 6 weeks to 18 months. One study utilized only a nutritional intervention (13) and one study applied only exercise intervention (25). Six studies performed nutritional intervention plus exercise intervention (8, 9, 14, 20, 21, 23). One study allied nutritional and exercise interventions to administration of thyroxine (22).

In 7 studies, a psychological intervention was added to the nutritional plus exercise intervention (10–12, 15, 17–19). One study performed the 3 interventions plus levothyroxine supplementation (26). One study performed psychological and exercise interventions (24) and 1 study used only a surgical intervention (16).

### 3.3. Nutritional interventions

Seventeen studies used nutritional interventions (8–15,, 17–24, 26). In many studies, the characteristics of the intervention were not detailed. The descriptions of the nutrition interventions performed included: dietary recommendations (11), meetings with dietician (20), diet list based on the ideal body weight (23), behavioral modification and diet plans (26). Four studies used a calorie restriction approach, with varying energy levels and macronutrient combinations (10, 13, 21, 22). Two studies used 1200 kcal/day (13, 21). One study adapted the caloric restriction to the subject’s weight, using 1,200, 1,400, or 1,600 kcal/day for the subjects with less than 50 Kg, 50-80 Kg, or more than 80 Kg of body weight, respectively (10). One study decreased the restriction throughout the duration of the intervention, using 600 (up to 1.5 month), 1,100 (1.5 to 6 months), or 1,400 kcal/day (6-12 months) (22). Nine studies reported the use of a nutrition education approach (8, 9, 12, 14, 15, 17–19, 24). Professionals in nutrition and dietetics performed sessions relaying information on food selection, diet, food habits, and food macronutrients composition and energetic densities. The sessions were directed to the children/adolescents only (14, 15, 17–19) or included also their parents (8) and school teachers (12). In two studies only the parents were addressed (9, 24).

### 3.4. Exercise interventions

Sixteen studies utilized physical exercise interventions. Eleven studies conducted supervised training sessions with no specific routine or duration, with varying types and intensities (9, 11, 12, 14, 15, 17–20, 24, 26), 1 involved two daily group endurance exercise sessions to improve aerobic performance, with a typical session lasting 60–90 min (10), and 3 performed aerobic exercise 3–5 times/week for at least 45–60 min (8), 30 min per day (23), or 90 min per day (21). One study involved either high- or moderate-intensity interval training for 45 min 3 times/week (25).

### 3.5. Psychological intervention

This type of intervention was used in 9 studies and included individual psychological care of the child/adolescent (10–12,17, 26) or of them and their family (15, 18, 19, 24). In the 2 studies detailing the psychological intervention, it consisted of techniques focusing on increasing self-esteem, responsibilities, and problem-solving strategies (24), relaxation techniques and breathing therapy (10).

## 4. Discussion

All the 19 studies included in this analysis, published between 1999 and 2020, achieved anthropometric recovery of the overweight/obese children/adolescents in response to the interventions, which, as depicted in Supplementary Table 1, varied largely with respect to the type and duration. Among the 17 studies utilizing nutritional interventions (8–15, 17–24, 26), 9 studies stated the use of a nutrition education approach (8, 9, 12, 14, 15, 17–19, 24). There was no mention about a distinction between nutrition education and nutrition counseling (27) in any of these 9 studies. One study (12) performed a nutrition intervention based on consultations focused on a “Motivational Interviewing”, aimed at stimulating behavioral change (28). Four studies utilized a 1-year lifestyle intervention called Obeldicks, in which the nutritional intervention consisted of individual coaching on the concept of prevention through an “optimized mixed diet”(15, 17–19). One study reported the use of an educational program in which normocaloric dietary guidelines were proposed to children and parents, based on the adoption of the Mediterranean diet and considering the dietary habits and age of the children (8). One study reported that “the dietary intervention consisted of 6 months of meetings with a dietician, where the participants received nutritional education, and information about food choices, diet, cooking and eating habits”(14). One study reported that an individual food plan was developed for each child/family, based on their specific needs and possibilities. They stated that: “The behavior change strategies used were motivational interviewing, goal setting, positive reinforcement, social support, and relapse prevention” (24). One study explained the nutrition intervention as follows: “Attempts to understand the causes of unhealthy habits and to obtain changes from the family; imply parents in these changes; obtaining the disappearance of junk foods from family shelves; decreasing the caloric density of cooking and the quantities served, without any prohibition except fried foods and chocolate spreads” (9).

Concerning the baseline levels (before intervention) of the thyroid hormones (fT3, fT4, TT3 or TT4), 6 studies did not report these data (13–16, 24, 26). Among the 13 studies in which this information was available, the majority (11 studies) reported no significant alterations in baseline levels, either in relation to the normality ranges (9–12, 17–22, 25) or in comparison to eutrophic individuals (8, 23), although one of these latter studies reported higher levels of fT4 in obese than in eutrophic girls (23) and the other study reported small percentages of subjects with levels of fT3 (17.9%) or fT4 (1.28%) above normal range (8). Only one study reported higher mean fT3 values in the obese than in the eutrophic subjects, although still in the normal range (19). These results show that the most common status of thyroid hormones is of levels in the normal range.

TSH levels at baseline were not reported in 5 studies (13–16, 24) while 2 studies selected only individuals with hyperthyrotropinemia (22, 26). Among the remaining 12 studies, the mean levels were normal in 9 studies (8–10, 12, 19–21, 23, 25), although some of these studies found mean values in the high normal range and highlighted the presence of elevated levels in variable percentages of their cohorts, namely 1.9% (10), 28.6% (21), 13.1% (9), 17.2% (20), 3.2% (8), and 17% (19). Elevated baseline levels of TSH were reported in 3 studies (11, 17, 18). These data demonstrate that the most common status of TSH among the studies analyzed fell into normal levels, although the finding of values in the high normal range was frequent.

Six studies reported correlations between body measures and hormone levels at baseline. Two studies reported a positive association of fT3 with BMI-SDS, or BMI/Age Z-score (8, 12) while another study found a positive association of fT3 and body weight and a negative association of fT4 and percentage body fat (13). In 3 studies, TSH correlated positively with BMI-SDS (8, 11) and BMI Z-score (9).

We searched other studies reporting levels of the hormones of the thyroid axis in children/adolescents with overweight/obesity. In one study, no differences were found in the levels of fT4 and TSH between children/adolescents with excess weight and the eutrophic ones (18). Many studies showed that these levels felt into the normal range, although a common finding was that they were higher than those of eutrophic children/adolescents, concerning TT3 (29), TT4 (30), and TSH (29–33), fT3 and fT4 (5).

Similar findings have been found in adults, with respect to fT3 (34) and TT4 (34, 35), i.e., levels in the normal range but higher than the eutrophic levels. There are also reports that the hormone levels were in the normal range but lower in obese than in eutrophic adults, concerning TSH (34–36), fT3 and fT4 (36).

Examining studies performing correlation analysis of hormone levels and body composition parameters in overweight/obese children/adolescents, we observed one study reporting no significant associations of fT3, fT4, and TSH levels with body composition parameters (37). In contrast, we found reports of a positive correlation between fT3 and BMI (5) and of a negative correlation of fT4 and BMI (30). There are also studies showing a positive correlation of TSH and body measures (5, 38). These latter results agree with the findings of the studies analyzed in this scoping review.

These findings are like some reports in adults, finding positive associations between BMI and TT3 (39) and fT3 (34, 36, 39–43), and a negative correlation with fT4 (43). With respect to TSH, the studies found achieved a negative (35) or positive (43–46) association of BMI and TSH.

Concerning the response to the interventions, 12 of the studies included in this scoping review reported a decrease in at least one thyroid hormone measured (TT3, TT4, fT3, fT4) in relation to the respective baseline values (8, 10, 12–17, 19–21, 24). Six studies did not find any significant change between the baseline and the post-intervention values (9, 11, 18, 23, 25, 26). One study did not report these data (22).

In relation to TSH, 12 studies reported a decrease after the intervention (9–12, 14, 16, 17, 19, 20, 22, 25, 26) and 5 studies showed no changes (13, 15, 18, 23, 24). It is important to point out that 2 of these studies (22, 26) included only subjects with hyperthyrotropinemia. In 2 studies, this information was not reported (8, 21).

The relation of hormonal levels and anthropometric recovery was evaluated in 6 studies by a correlation analysis. Positive associations were found between delta of fT3 and body weight, BMI-SDS, fat mass, and percentage of fat mass (10), fT3 and BMI Z-score (12, 14) and TT3 and BMI (16). TT4 correlated positively with BMI Z-score (15) while delta fT4 correlated negatively with percentage of fat mass (10). TSH correlated positively with BMI-SDS (11), BMI Z-score (14) and BMI (16). Studies performed in adults submitted to multidisciplinary interventions to treat obesity corroborate the above results, as they have found positive associations of fT3 or TT3 with BMI and body weight (39, 47, 48) and of TSH with body weight (48–51). However, we found one study in which body and fat mass losses were not accompanied by changes in TSH levels (52).

The main purpose of this scoping review was to ascertain whether the hormones of the thyroid axis vary during weight loss in overweight/obese children/adolescents. The examination of the 19 selected studies allowed us to conclude that most of the results pointed to the absence of elevated levels at baseline, in agreement with a previous review (5). Also, most of the studies showed that the changes in body composition parameters in response to the multidisciplinary interventions correlated positively with fT3, TT3, or TSH. Further studies are necessary to elucidate the relevance of the variations in hormone levels to the establishment of overweight/obesity and to the recovery from these conditions in children/adolescents. With respect to fT4 and TT4, the most common finding was of unchanged levels and hence, no significant association with weight loss. Importantly, the response to the intervention has even been found to not be affected by fT4 supplementation.

## Data availability statement

The original contributions presented in this study are included in the article/Supplementary material, further inquiries can be directed to the corresponding author.

## Author contributions

CU and ER: methodology. CU, ER, AC, and MF: review per peers. CU, AP, FT, and ER: formal analysis. CU, AP, FT, MT, AS, and ER: writing—original draft preparation. CU, ER, and MF: writing—review and editing. All authors have read and agreed to the published version of the manuscript.
