# Supplementary material for: Thyroid axis hormones and anthropometric recovery of children/adolescents with overweight/obesity: A scoping review
Source: Front Nutr. 2023 Jan 13;9:1040167. doi: 10.3389/fnut.2022.1040167 (PMC9880327; doi:10.3389/fnut.2022.1040167)
Supplement: Supplementary file 1 [file Table_1.docx]

**Table 1.** Summary of the characteristics and results of the 19 studies included in the analysis.

| **Reference** | **Sample (Age)** | **Groups/Gender** | **Intervention**  **type and duration** | **Effect of Intervention*** | **Hormones** | | **Authors’ conclusion** | **Our observation** |
| --- | --- | --- | --- | --- | --- | --- | --- | --- |
|  |  |  |  |  | **Baseline** | **After intervention** |  |  |
| **Studies allowing intra-group comparisons** | | | | | | | | |
| Martins et al. 2020 (8) | School 1: 73  School 2: 103  (8-11y) | School 1: 73 OW/ OB (BMI ≥ 1 Z-score)  27 M; 46 F  School 2: 103 OW/ OB (BMI ≥ 1 Z-score)  51 M; 52 F | School 1: Nutrition education workshops at school (10 months) followed by outpatient clinical care and nutritional counseling (16 months)  School 2: Nutrition education, supervised physical activity and reflective meetings at school (16 months) followed by outpatient clinical care and nutritional counseling (16 monts) | BMI Z-score decreased in both schools | **fT3:** both schools had normal levels  (School 2: 8% above normal range).  School 2 levels lower than School 1  **fT4:**  normal levels in both schools.  School 2 levels lower than School 1  **TSH**: normal levels in both schools.  School 2 levels lower than School 1 | **fT3:** decreased levels in both Schools.  School 1 levels lower than School 2  **FT4**: decreased levels in both schools.  School 1 levels lower than School 2  **TSH**: decreased levels in School 1 | ﻿The decrease in delta BMI/Age was similar in both schools ﻿and was accompanied by a decrease in TSH and fT3 concentrations | The different multidisciplinary interventions were effective in reducing BMI, fT3, fT4 and TSH |
| Lass et al. 2020 (9) | 28  (10.2 ± 2.2 y) | 28 OW  14 M; 14 F  ﻿(BMI > 90th percentile) | Lifestyle intervention  (physical exercise, nutrition education, and psychological therapy)  (1 year) | BMI Z-score decreased* | **fT3**: normal range  **fT4**: normal range  **TSH:** high range | **fT3**: decreased  **fT4**: unchanged  **TSH**: decreased | Thyroid volume correlates positively to weight status in childhood obesity. Change reverts after weight loss independently of thyroid function parameters | Weight loss induced by multidisciplinary intervention for 1 year decreased fT3 and TSH levels |

| Abasi et al. 2020 (10) | 16 (15 – 17y) | 16 OW/OB girls (No information on criteria) | Moderate- or High-intensity interval training  (MIIT or HIIT)  (3 months) | BMI Z-score decreased in both groups* | **fT4**: normal range  **TSH**: normal range | **fT4:** decreased only in HIIT  **TSH:** decreased only in HIIT | Pituitary-thyroid function is more sensitive to training intensity than training duration | Although both MIIT and HIIT decreased BMI similarly, only HIIT was effective in lowering fT4 and TSH |
| --- | --- | --- | --- | --- | --- | --- | --- | --- |
| Kiortsis et al.  1999 (11) | 64  (10 – 14y) | 64 OB (RBW>120%)  22 M; 42 F | Caloric restriction, including a 24h recall method  (6 weeks) | BMI decreased* | Baseline values not reported | **TT3:** decreased | The decline in TT3 levels seems to play an important role in the decrease of RMR in children | TT3 levels decreased along with weight loss |
|  |  |  |  |  |  | **TT4:** unchanged |  |  |
|  |  |  |  |  |  | **TSH:** unchanged |  |  |
| Rijks et al. 2017 (12) | 330  (2.6 – 18.9y) | 66 OW (BMI Z-score >75<90), 148 OB (BMI Z-score=90<97), 115 MO (≥97)  142 M; 188 F  99 out of 330 children were evaluated 1-year post-intervention | Tailored lifestyle intervention (nutritional education and physical activity  (1 year)  Clinical assessment 1-year post-intervention | BMI Z-score decreased* | **fT4:** no differences among the subgroups OW, OB and MO | **fT4:** decreased in children with decreased BMI Z-score (n = 62), but unchanged in children with increased BMI Z-score (n = 37) | In OW and OB, TSH is positively associated with CVD markers. Changes in TSH are also associated with changes in lipid concentrations in children showing weight loss | fT4 levels decreased along with weight loss |
|  |  |  |  |  | **TSH:** no differences among the subgroups OW, OB and MO | **TSH:** unchanged |  |  |
| Aeberli et al. 2010 (13) | 206  (10 – 18y) | 206 OB (BMI-SDS >98 percentile)  119 M; 87 F  197 OB completed the intervention | Hypocaloric diet, physical activity, and ﻿psychological treatment  (8 weeks) | BMI-SDS decreased* | **fT3**: normal range | **fT3:** decreased | TSH concentrations tend to be higher in OB | fT3 and TSH levels decreased along with weight loss |
|  |  |  |  |  | **fT4:** normal range | **fT4:** unchanged |  |  |
|  |  |  |  |  | **TSH:** normal range (52% in the high normal range and 1.9% had HTTR) | **TSH:** decreased (but 44% in the high normal range and 0.5% had HTTR) |  |  |

| Shalitin et al. 2009 (14) | 207  (5 – 18y) | 207 OB (BMI-SDS >95 percentile)  97 M; 110 F  142 subjects completed the intervention | Hypocaloric diet  (12 weeks) | BMI-SDS decreased in 125 subjects* | **TT3:** normal range | **TT3:** not reported | In OB, high TSH levels with normal fT4 levels appears to be frequent | No significant differences regarding fT4 and TSH between the children showing decreased BMI-SDS and those showing no BMI-SDS changes |
| --- | --- | --- | --- | --- | --- | --- | --- | --- |
|  |  |  |  |  | **fT4:** normal range | **fT4:** Δ 0.18 ± 1.9 in the 125 children with decreased BMI-SDS;    Δ -0.6 ± 1.97 in the 16 children with no BMI-SDS changes |  |  |
|  |  |  |  |  | **TSH:** 161 subject showed normal range levels; 46 subjects showed levels above the normal range | **TSH**: Δ –0.28 ±1.12 in the 125 children with decreased BMI-SDS;  0.06 ± 2.13 in the 16 children with no BMI-SDS changes |  |  |
| Bas et al. 2013 (15) | 150  (3 – 17y) | 150 OB (BMI Z-score >percentile 95)  67 M; 83 F  Only 21 OB who had TSH levels above the normal range at baseline were evaluated after the intervention | Nutrition education and physical activity  (6 months) | BMI Z-score decreased | Baseline values not reported | **fT3:** decreased | TSH and fT3 levels are significantly increased in childhood obesity | In OB with high TSH levels,fT3 and TSH levels decreased along with weight loss |
|  |  |  |  |  |  | **fT4:** unchanged |  |  |
|  |  |  |  |  |  | **TSH:** decreased |  |  |
|  |  |  |  | BMI Z-score unchanged |  | **fT3:** unchanged |  |  |
|  |  |  |  |  |  | **fT4:** unchanged |  |  |
|  |  |  |  |  |  | **TSH:** unchanged |  |  |

| Wolters et al. 2013 (16) | 477  (10.6 ± 2.7y) | 477 OB (BMI-SDS >97 percentile)  219 M; 258 F | Lyfestyle intervention  (physical exercise, nutrition education, and psychological therapy)  (1 year) | BMI-SDS decreased * | **fT3:** normal range | **fT3:** unchanged | Moderately increased TSH and fT3 concentrations in OB, which normalized after substantial weight loss | No changes in fT3, fT4 and TSH levels along with weight loss |
| --- | --- | --- | --- | --- | --- | --- | --- | --- |
|  |  |  |  |  | **fT4:** normal range | **fT4:** unchanged |  |  |
|  |  |  |  |  | **TSH:** High range | **TSH:** unchanged |  |  |

| Licenziati et al. 2019 (17) | 96  (4.7 – 16.4y) | 96 OB (BMI-SDS >75 percentile)  49 M; 47 F | Dietary recommendations, physical activity, and behavioral strategies  (0.8 ± 0.3 year) | BMI-SDS decreased * | **fT4:** normal range | **fT4:** unchanged | The alterations of thyroid function and structure in OB are reversible after weight loss | OB with alterations of thyroid structure (volume, echogenicity, homogeneity of parenchima) showed decreased TSH levels along with weight loss |
| --- | --- | --- | --- | --- | --- | --- | --- | --- |
|  |  |  |  |  | **TSH:** High range  - TSH levels exceeded the normal range in 83.3% of the OB | **TSH:** decreased  - TSH levels exceeded the normal range in 57.3% of OB |  |  |
| Bouglé et al. 2014 (18) | 528  (4.1 – 17.9y) | 528 OW + OB (BMI Z-score >2 SDS)  238 M; 290 F  79 subjects completed the intervention | Nutritional education and physical activity  (52 ± 15 weeks) | BMI Z-score decreased | **fT3:** normal range | **fT3:** unchanged | Increased TSH may be predictive of decreased insulin resistance; fT4 was associated with a low metabolic risk. Changes in thyroid function could protect against obesity-associated metabolic diseases | Non-significant decrease of fT3, fT4, TSH and BMI Z-score |
|  |  |  |  |  | **fT4:** normal range | **fT4:** unchanged |  |  |
|  |  |  |  |  | **TSH:** normal range levels, but 69 showed levels above the normal range | **TSH:** in the group with normal TSH at baseline, 26 and 42 subjects showed decrease and increased TSH levels, respectively. TSH decreased in all patients with initial values above the normal range |  |  |
| Radetti  et al. 2012 (19) | 72  (8 – 14y) | 72 OW/OB (BMI-SDS >85 percentile)  41 M; 31 F | Meetings with dietician and instructions on physical activity at 3-month intervals (1.8 ± 1.0 year) | BMI-SDS decreased | **fT3:** normal range | **fT3:** decreased | A yet slightly reduced body fat can be followed by an improvement in biochemical parameters such as TSH and fT3 | fT3 and TSH levels decreased along with a non-significant weight loss |
|  |  |  |  |  | **fT4:** normal range | **fT4:** unchanged |  |  |
|  |  |  |  |  | **TSH:** normal range (17.2% high range) | **TSH:** decreased. 6.2% of the children showed a TSH above the normal range |  |  |

| **Studies conducted with** **comparisons between excess weigth and euthrophic groups** | | | | | | | | |
| --- | --- | --- | --- | --- | --- | --- | --- | --- |
| Marras et al. 2010 (20) | 520 (3.7 – 17.9y) | Intervention group  468 OB (BMI-SDS >95 percentile)  213 M; 255 F  Control Group  52 EUT  24 M; 28 F | Educational program: Dietary guidelines and physical activity  (6 months) | BMI-SDS decreased in the OB group | **fT3:** no difference (17.9% above the normal range) | fT3 and fT4 normalized in 63% of the patients who showed abnormal concentrations at baseline | In OB, an increased fT3 concentration is the most frequent thyroid function abnormality. Serum fT3 and TSH correlate with BMI-SDS. Moderate weight loss frequently restores these abnormalities | fT3 levels were higher in only 17.9% of OB. 63% of OB who had abnormal thyroid hormone levels presented normalization of these hormones along with weight loss |
|  |  |  |  |  | **fT4:** no differences (1.28% above normal range) |  |  |  |
|  |  |  |  |  | **TSH:** no differences (3.2% above normal range) |  |  |  |

| Cayir et al. 2014 (21) | 109  (5 – 18y) | Intervention group:  85 (OW: 28; OB: 29; MO: 29) (No information on criteria)  43 M; 42 F  Control Group:  24 EUT  13 M; 11 F | Diet list based on the ideal body weight. Recommendation of 30 min aerobic exercise/day  (6 months) | BMI of OW, OB and MO subgroups decreased * | **Between-groups comparison**  **(n = 50)** | | Normal thyroid function in OB, not influenced by a prolonged period of caloric restriction | fT4 levels were higher in OB girls than in EUT girls, reaching normal levels along with weight loss induced by caloric restriction |
| --- | --- | --- | --- | --- | --- | --- | --- | --- |
|  |  |  |  |  | **fT3:** no differences |  |  |  |
|  |  |  |  |  | **fT4:** no differences between boys, but OB girls had higher levels than EUT girls |  |  |  |
|  |  |  |  |  | **TSH:** no differences |  |  |  |
|  |  |  |  |  | **In-group comparison (n = 36)** | |  |  |
|  |  |  |  |  | **fT3:** unchanged  **fT4:** no changes among the boys. OB girls showed decreased levels (became like the EUT girls)  **TSH:** unchanged |  |  |  |
|  |  |  |  |  |  |  |  |  |
|  |  |  |  |  |  |  |  |  |

| Reinehr et al. 2006 (22) | 317  (9.5 – 12y) | Intervention group:  246 OB (BMI Z-score >97 percentile)  109 M; 137 F  49 OB completed the intervention  Control group:  71 EUT  (30 M; 41 F)  (Information about the treatment or intervention performed for this group is not described) | Lyfestyle intervention  (physical exercise, nutrition education, and psychological therapy)  (1 year) | BMI Z-score decreased with substantial weight loss | **fT3**: OB had higher levels than EUT (normal range) | **fT3**: OB with substantial weight loss showed decreased levels | fT3 and TSH were moderately increased in OB and weight loss led to a reduction. The elevation of these hormones seems to be rather a consequence of obesity than a cause of obesity | ﻿ Substantial weight loss decreased fT3 and TSH |
| --- | --- | --- | --- | --- | --- | --- | --- | --- |
|  |  |  |  |  | **fT4**: no differences (normal range)  **TSH**: OB had higher levels than EUT (17% above normal range) | **fT4**: unchanged  **TSH**: OB with substantial weight loss showed decreased levels |  |  |
|  |  |  |  |  |  |  |  |  |

| Reinehr et al. 2002 (23) | 225  (4.5 – 16y) | Intervention group:  118 OB (BMI Z-score >97 percentile)  63M; 55 F  - 68 OB completed the intervention  Control group:  107 EUT  (61 M; 46 F)  (No information about intervention in the control group) | Physical exercise, nutrition education, and psychological therapy  (12 months) |  |  |  | **TT3, TT4 and TSH levels were moderately increased in OB at baseline**. A normal energy diet induces a long-term decrease in the peripheral thyroid hormones as opposed to TSH | TT3, TT4 and TSH levels moderately increased in OB than in controls  TT3 and TT4 levels decreased along with intervention-induced weight loss in OB |
| --- | --- | --- | --- | --- | --- | --- | --- | --- |
|  |  |  |  | BMI Z-score decreased  (n=55) | Baseline values not reported | **TT3:** decreased |  |  |
|  |  |  |  |  |  | **TT4:** decreased |  |  |
|  |  |  |  |  |  | **TSH:** unchanged |  |  |
|  |  |  |  |  |  |  |  |  |
| Butte et al. 2015 (24) | 16  (12 – 17y) | Intervention group:  11 MO (BMI >50 or ≥40 with comorbidities)  3 M; 2 F  Control group:  5 MO who rejected bariatric surgery | Roux-en-Y gastric bypass surgery (RYGB).  The subjects were reevaluated 12 months after surgery | BMI decreased after RYGB* | Baseline values not reported | **TT3:** decreased | Energy adaptations that occur in adolescents following RYGB possibly involves TT3 mediation | TT3 and TSH levels decreased along with BMI decrease induced by RYGB |
|  |  |  |  |  |  | **fT3:** unchanged |  |  |
|  |  |  |  |  |  | **TT4:** unchanged |  |  |
|  |  |  |  |  |  | **fT4:** unchanged  **TSH:** decreased |  |  |
|  |  |  |  |  |  |  |  |  |

| **Studies involving thyroxine supplementation** | | | | | | | | |
| --- | --- | --- | --- | --- | --- | --- | --- | --- |
| Eliakim et al. 2006 (25) | 41 OB (5 – 17y) with HTTR | Intervention group:  26 OB (BMI >85 percentile), with HTTR,  with thyroxine supplementation  Control group:  15 OB, with HTTR, without thyroxine supplementation | Caloric restriction and exercise program for 12 months followed by thyroxine supplementation (1-2 μg/kg) for 6 months | BMI decreased in both supplemented and not supplemented groups | **fT4:** normal range | **fT4:** no differences between supplemented and non-supplemented groups | HTTR is relatively common in OB. TSH levels returned to normal in the majority of patients even without thyroid hormone administration. No beneficial effects on body weight and BMI were found in treated subjects, suggesting that thyroid substitution is not necessary in most cases | TSH levels decreased independent of thyroxine supplementation |
|  |  |  |  |  | **TSH:** HTTR | **TSH:** decreased in both supplemented and not supplemented groups |  |  |
| Kumar et al. 2019 (26) | 51 OB with HTTR  (6 – 12y) | Intervention group:  26 OB (BMI Z-score >3) with levothyroxine supplementation  Control group:  25 OB without levothyroxine supplementation | Behavioral modification and a diet and physical activity plan, with or without levothyroxine supplementation (0.5 μg/kg/day)  (6 months) | BMI Z-score decreased in both supplemented and not supplemented groups | **TSH:** HTTR | **TT3:** no differences between groups and versus baseline | Supplementation of levothyroxine during weight management interventions, and should not be prescribed to children with obesity associated thyroid dysfunction | Supplementation with levothyroxine has no effect on weight loss |
|  |  |  |  |  |  | **TT4:** no differences between groups and versus baseline |  |  |
|  |  |  |  |  |  | **TSH:** decreased in both groups. No differences between groups. |  |  |

* p<0.05 versus baseline according to the effect of the intervention in relation to the body mass index.

T3 = triiodothyronine; T4 = thyroxine; TSH = thyrotropin; TT3 = total T3; TT4 = total T4; fT3 = free T3; fT4 = free T4

M = male; F = female; BC = body composition; EUT = eutrophic; OW = children with overweight; OB: children with obesity; MO = children with morbid obesity; MIIT = Moderate-intensity interval training; HIIT: High-intensity interval training; HTTR = hyperthyrotropinemia; RMR = resting metabolic rate.

RBW = Relative body weight: child's weight divided by the expected weight for height and multiplied by 100.x
